# Relativistic Consistency of Nonlocal Quantum Correlations

**DOI:** 10.3390/e26070548

**Published:** 2024-06-27

**Authors:** Christian Beck, Dustin Lazarovici

**Affiliations:** Humanities and Arts Department, Technion–Israel Institute of Technology, Haifa 3200003, Israel; dustin@technion.ac.il

**Keywords:** relativistic quantum theory, nonlocality, no-signaling, local commutativity, sequential quantum measurements, time-order (in)dependence

## Abstract

What guarantees the “peaceful coexistence” of quantum nonlocality and special relativity? The tension arises because entanglement leads to locally inexplicable correlations between distant events that have no absolute temporal order in relativistic spacetime. This paper identifies a relativistic consistency condition that is weaker than Bell locality but stronger than the no-signaling condition meant to exclude superluminal communication. While justifications for the no-signaling condition often rely on anthropocentric arguments, relativistic consistency is simply the requirement that joint outcome distributions for spacelike separated measurements (or measurement-like processes) must be independent of their temporal order. This is necessary to obtain consistent statistical predictions across different Lorentz frames. We first consider ideal quantum measurements, derive the relevant consistency condition on the level of probability distributions, and show that it implies no-signaling (but not vice versa). We then extend the results to general quantum operations and derive corresponding operator conditions. This will allow us to clarify the relationships between relativistic consistency, no-signaling, and local commutativity. We argue that relativistic consistency is the basic physical principle that ensures the compatibility of quantum statistics and relativistic spacetime structure, while no-signaling and local commutativity can be justified on this basis.

## 1. Introduction

What guarantees the “peaceful coexistence” [1] of quantum nonlocality and special relativity? The condition generally emphasized is that the (marginal) probabilities for any local measurement *A* must be independent of other measurements *B* occurring at spacelike separation:

(1) $$P\left(A=\alpha \right)\overset{!}{=}P\left(A=\alpha |B\;\mathrm{was}\;\mathrm{measured}\right)=\sum_{\beta }P\left(A=\alpha |B=\beta \right)\;P\left(B=\beta \right).$$

This is not to be confused with Bell’s *locality* condition, which requires that correlations between spacelike separated events can be explained (“screened off”) by conditionalizing on suitable variables λ in their past:

(2) P(A=α∧B=β∣λ)=P(A=α∣λ)P(B=β∣λ),

Bell’s theorem (see [2], Chs. 2, 16, 24) and the empirical violation of Bell inequalities (e.g., [3]) establish that (2) is violated for certain nonlocal correlations that arise from joint measurements on entangled systems.

Equation (1) is known as the *no-signaling* condition. The idea is that while an agent cannot control the random outcomes of a quantum experiment to exploit the violations of (2), they can decide which (if any) measurement *B* to perform and thus, if (1) were violated, communicate superluminally by influencing the statistics of the distant measurement *A* (see Section 3.3.2 for more details).

But why exactly is *no-signaling* (as opposed to Bell-locality) necessary and/or sufficient to avoid head-on contradictions between special relativity and the statistical predictions of quantum theory? By and large, two lines of argument are pursued in the literature.

The first is quasi-axiomatic. It has become standard to refer to the light-cone structure of relativistic spacetime as its *causal* structure, and presentations of the theory often include a basic principle to the effect that events can be causally influenced only by events in or on their past light-cones. Against this backdrop, there are attempts to argue that a violation of (1), but not a violation of (2), amounts to a “causal influence” (See, e.g., [1,4,5,6,7]. In some cases, a distinction is made between violations of *parameter independence* and *outcome independence* in the context of Bell’s theorem. For standard quantum theory, where λ=ψ, parameter independence is equivalent to no-signaling. For a critical discussion of this distinction, see [8]).

Aside from the fact that we find these attempts uncompelling, the problem with the approach is two-fold. First, it is unnecessary to include such a causality principle among the fundamental axioms of relativity theory. Special (and also general) relativity can be understood as a theory about the geometric structure of spacetime without invoking causal notions. Second, it is advisable to do so since centuries of philosophical debate show how difficult and contentious the analysis of causal notions is. More importantly, in any concrete physical sense, the compatibility of relativity and quantum theory can hardly depend on which philosophical analysis one endorses, say, on whether one accepts Reichenbach’s *common cause principle* or insists on an interventionist theory of causation (see [9] for an overview of these debates in the context of quantum nonlocality).

The structure of Minkowski spacetime *per se* does not rule out faster-than-light signaling. Maudlin [10] gives an extensive account of possible physical processes that are superluminal but compatible with relativistic spacetime structure. Even tachyons can be easily implemented in a relativistic theory [11,12]. Strictly speaking, the relativity principle—i.e., the equivalence of all inertial Lorentz frames—implies only the existence of an *invariant* velocity, not of a *maximal* one. This is to say that while “signals” are not necessarily the kind of things that literally propagate through spacetime, even the superluminal propagation of matter cannot be excluded a priori.

The other (slightly better) argument for (1) contends that special relativity does not rule out superluminal signaling by postulate but by entailing that it would give rise to causal paradoxes (see, e.g., [13,14,15,16]). This is due to the absence of an absolute temporal order between spacelike separated events. Moving at suitable (subluminal) velocities relative to each other, Alice could receive a signal from Bob and send a superluminal response that reaches Bob before he sent out his original signal. The worry is now that the two agents could agree on a communication protocol such as the following: Alice sends her signal if and only if she receives one from Bob. Bob sends his signal if and only if he does not receive one from Alice. This results in a grandfather-like paradox (in the present context sometimes referred to as *Tolman’s paradox* after [17]).

One reason why such arguments remain unsatisfactory is that they are based on strong but possibly fallible intuitions about free agency on the macro level. Even if Alice and Bob were replaced by purely mechanical devices, the thought experiment assumes our ability to build and set up such devices and ensure their reliable operation. Nature, however, could always find ways to enforce its logical consistency, e.g., by making communication devices malfunction or having all funding requests for superluminal communication experiments denied. In less anthropomorphic terms, the point is that if a theory has microscopic states that would allow superluminal signaling and we can conceive of macroscopic conditions under which superluminal signaling results in inconsistent histories, it does not follow that the theory allows for microscopic states that realize such paradoxical macro-conditions. Inconsistent histories may simply not arise as solutions of the dynamical theory once all relevant variables—including those describing the measurement devices, the experimentalists, etc.—are taken into account (for an instructive analysis in the context of Wheeler–Feynman electrodynamics, see [18]).

That the causal-paradox argument may simply not go through is one thing. The more basic issue is that the concept of “signaling” is somewhat anthropocentric to begin with, which calls into question its suitability as a “fundamental” physical principle. As John Bell said,

Do we then have to fall back on ‘no signaling faster than light’ as the expression of the fundamental causal structure of contemporary theoretical physics? That is hard for me to accept. For one thing we have lost the idea that correlations can be explained, or at least this idea awaits reformulation. More importantly, the ‘no signaling…’ notion rests on concepts which are desperately vague, or vaguely applicable. The assertion that ‘we cannot signal faster than light’ immediately provokes the question:

Who do we think *we* are?

[2] (p. 245)

The following discussion takes a fresh look at the following question: What is a necessary condition for avoiding contradictions between nonlocal quantum correlations and the special-relativistic spacetime structure? We will identify a simple criterion of *relativistic consistency* that is stronger than the no-signaling condition (1) but whose justification does not rely on causal principles or anthropocentric reasoning. Instead, the rationale is simply the following:

1. Relativistic spacetime structure includes no absolute temporal order between spacelike separated events.
2. Statistical predictions for joint local measurements must be consistent across Lorentz frames that disagree on the order of the measurements (with respect to coordinate time).

While this line of argument may seem obvious, we are only aware of a single publication by other authors that offers a similar reasoning, namely [16].

One can object that the concept of “measurements” is also vague and anthropocentric. Indeed, we believe that a satisfactory reconciliation of nonlocality and relativity ultimately requires a quantum theory that solves the measurement problem and explains nonlocal correlations in terms of a coherent ontology of matter. (For promising approaches, see e.g., [19,20,21,22]). Such a theory would no longer involve “measurements” as a basic concept, and relativistic consistency would simply pertain to the distribution of matter in space and time. But the scope of this paper is much more modest (and uncontroversial). It concerns the compatibility of special relativity and the statistical predictions of the quantum formalism wherever the latter applies and however it may be grounded in a more complete or fundamental theory. To this end, we will speak about “measurements” and “measuring devices”. This can be taken literally if one wants to stick to the minimal consensus about the validity of the quantum formalism. It does not have to be taken literally, though. “If the theory is to apply to anything but highly idealised laboratory operations…, ‘measurement-like’ processes are going on more or less all the time, more or less everywhere” [2] (p. 216). Our considerations will apply just as well to “measurement-like processes” that need not involve any human agency.

## 2. Quantum Probabilities

### 2.1. Ideal Measurements

In the following sections, we will derive and discuss our main results in the framework of ideal quantum measurements (the kind most familiar from textbook quantum mechanics). Since our objective is to analyze two measurements at spacelike separation, we shall refer to an “*A*-measurement” and a “*B*-measurement” (for more than two measurements, the discussion generalizes in the obvious way). Their possible outcomes are denoted by α and β, respectively. In the ideal measurement scheme, they range over some discrete subsets of real numbers and are associated with orthogonal projections $P_{\alpha }^{A}$ and $P_{\beta }^{B}$ acting on some Hilbert space H.

Via the spectral theorem, the outcomes and corresponding projections can be encoded in self-adjoint operators (*observables*) $A=\sum_{\alpha }\alpha P_{\alpha }^{A}$ and $B=\sum_{\beta }\beta P_{\beta }^{B}$. If the measurements are performed on a system with initial state ψ∈H, the outcome probabilities are given by the Born rule

(3) $$P^{\psi }\left(A=\alpha \right)=∥P_{\alpha }{\;\psi ∥}^{2}\;\;\mathrm{and}\;\;P^{\psi }\left(B=\beta \right)={∥P_{\beta }\;\psi ∥}^{2}$$

and final (post-measurement) states by

(4) $$\psi_{\alpha }:=\frac{P_{\alpha }\;\psi }{∥P_{\alpha }\;\psi ∥}\;\;\mathrm{and}\;\;\psi_{\beta }:=\frac{P_{\beta }\;\psi }{∥P_{\beta }\;\psi ∥}.$$

Such ideal measurements with their (overly) simple projection postulate allow us to introduce and discuss the relevant concepts in the most comprehensible manner. Our results then generalize straightforwardly to the modern operationalist formalism with positive operator-valued measures (POVMs), density operators, and completely positive mappings. This is carried out in Section 4.

### 2.2. Time-Order Dependency

A prominent feature of quantum formalism is that it does not provide joint probability distributions for observables that cannot be measured simultaneously [23]. However, if such measurements are performed successively, quantum theory is perfectly capable of predicting joint probabilities for their outcomes, only that the probabilities will depend on the *order* in which the measurements occur.

We can demonstrate this time-order dependency with ideal spin measurements. Let $H≅C^{2}$ and $\sigma_{i}$ with i=x,y,z be the Pauli matrices with eigenstates defined by the relations $\sigma_{i}{\;|↑}_{i}{\rangle =+1|↑}_{i}\rangle$ and $\sigma_{i}{\;|↓}_{i}{\rangle =-1|↓}_{i}\rangle$, respectively. The probabilities associated with an ideal measurement of the spin component *i* are given by the two projections P↑i=↑i↑i↑i↑i and P↓i=↓i↓i↓i↓i, which define a PVM (projection-valued measure). One might now ask for joint probabilities, e.g., the probability

(5) $$P^{\psi }\left(\sigma_{x}=+1\wedge \sigma_{z}=-1\right)$$

that successive measurements will find *x*-*spin up* and *z*-*spin down* if two particles are initially prepared in the state ψ∈H. But (5) is not well-defined or, rather, underdetermined. Since $\sigma_{x}$ and $\sigma_{z}$ are not simultaneously measurable, one must specify the order in which the two spin measurements are carried out.

Always well-defined are conditional probabilities such as

(6) $$P^{\psi }\left(\sigma_{z}=-1|\sigma_{x}=+1\right),$$

meaning the probability of obtaining $\sigma_{z}=-1$, given that a preceding measurement on the initial state ψ had the outcome $\sigma_{x}=+1$ (and assuming that the free time evolution between the two measurements can be neglected, which we shall always assume for simplicity). According to the quantum formalism, this probability is obtained from the Born rule using the final state of the first measurement, viz. $\psi_{↑}^{x}:=\frac{P_{↑}^{x}\;\psi }{∥P_{↑}^{x}\;\psi ∥}$, as the initial state of the subsequent $\sigma_{z}$-measurement:

(7) Pψσz=−1∣σx=+1=Pψ↑x(σz=−1)=P↑xψP↓zP↑xψP↓zP↑xψP↑xψP↑xψP↑xψP↑xψP↑xψ=ψP↑xP↓zP↑xψP↑xP↓zP↑xψψψP↑xψP↑xψψ.

These conditional probabilities suggest that we introduce the notation

(8) $$P^{\psi }\left(\sigma_{x}=+1\vec{\wedge }\sigma_{z}=-1\right):=P^{\psi }\left(\sigma_{z}=-1|\sigma_{x}=+1\right)\;P^{\psi }\left(\sigma_{x}=+1\right)$$

for the joint probability when the measurement of the *x*-spin component comes first, and

(9) $$P^{\psi }\left(\sigma_{x}=+1\overset{↼}{\wedge }\sigma_{z}=-1\right):=P^{\psi }\left(\sigma_{x}=+1|\sigma_{z}=-1\right)\;P^{\psi }\left(\sigma_{z}=-1\right)$$

when the measurement of the *z*-component precedes the *x*-spin measurement.

To compute the probabilities (8) and (9) for, say, the initial state $\psi ={|↑}_{x}\rangle$, we use the relations ${|↑}_{x}\rangle =\frac{1}{\sqrt{2}}\left({|↑}_{z}{\rangle +|↓}_{z}\rangle \right)$ and ${|↓}_{z}\rangle =\frac{1}{\sqrt{2}}\left({|↑}_{x}{\rangle -|↓}_{x}\rangle \right)$. Equation (8) now yields

(10) Pψ(σx=+1∧⇀σz=−1)=Pψσz=−1∣σx=+1Pψσx=+1==ψP↑xP↓zP↑xψP↑xP↓zP↑xψψψP↑xψP↑xψψψP↑xψP↑xψψ=〈↑x|P↑xP↓zP↑x|↑x〉=〈↑x|P↓z|↑x〉==12〈↑z|+〈↓z|P↓z|↑z〉+|↓z〉=12.

On the other hand, Equation (9) evaluates as

(11) Pψ(σx=+1∧↼σz=−1)=Pψσx=+1∣σz=−1Pψσz=−1==ψP↓zP↑xP↓zψP↓zP↑xP↓zψψψP↓zψP↓zψψψP↓zψP↓zψψ=〈↑x|P↓zP↑xP↓z|↑x〉==12〈↑z|+〈↓z|P↓zP↑xP↓z|↑z〉+|↓z〉=12〈↓z|P↑x|↓z〉==14〈↑x|−〈↓x|P↑x|↑x〉−|↓x〉=14.

In summary, we have

$$P^{\psi }\left(\sigma_{x}=+1\vec{\wedge }\sigma_{z}=-1\right)=\frac{1}{2}$$

while

$$P^{\psi }\left(\sigma_{x}=+1\overset{↼}{\wedge }\sigma_{z}=-1\right)=\frac{1}{4}$$

and thus,

(12) $$P^{\psi }\left(\sigma_{x}=+1\vec{\wedge }\sigma_{z}=-1\right)\neq P^{\psi }\left(\sigma_{x}=+1\overset{↼}{\wedge }\sigma_{z}=-1\right).$$

Of course, this has to do with the non-commutativity of the operators $\sigma_{x}$ and $\sigma_{z}$, but we will return to this topic later.

The fact that joint probability distributions for the outcomes of successive measurements can depend on the temporal order of the measurements lies behind many of the features of quantum probabilities that are often described as “non-classical”. In particular, we found

(13) $$P^{\psi }\left(\sigma_{z}=-1|\sigma_{x}=+1\right)\;P^{\psi }\left(\sigma_{x}=+1\right)\neq P^{\psi }\left(\sigma_{x}=+1|\sigma_{z}=-1\right)\;P^{\psi }\left(\sigma_{z}=-1\right),$$

while it seems to follow from the definition of conditional probability that both sides are equal and correspond to $P^{\psi }\left(\sigma_{x}=+1\wedge \sigma_{z}=-1\right)=P^{\psi }\left(\sigma_{z}=-1\wedge \sigma_{x}=+1\right)$. In fact, (13) is neither mysterious nor in contradiction with standard probability theory if one appreciates that measurements change the state of the measured system in ways that can affect the statistics of subsequent measurements. As a consequence, the conditional probabilities on the left- and right-hand side of (13) correspond to different joint distributions (those on the left- and right-hand side of (12), respectively).

The possible time-order dependence of joint distributions motivates the following definition for general ideal quantum measurements.

**Definition** **1** (Time-dependent joint probabilities)**.** *We denote joint outcome probabilities for sequential measurements A and B, which can depend on the time order of the measurements, by*

(14) $$P^{\psi }\left(A=\alpha \overset{↼}{\wedge }B=\beta \right):=P^{\psi }\left(A=\alpha |B=\beta \right)\;P^{\psi }\left(B=\beta \right)$$

*and*

(15) $$P^{\psi }\left(A=\alpha \vec{\wedge }B=\beta \right):=P^{\psi }\left(B=\beta |A=\alpha \right)\;P^{\psi }\left(A=\alpha \right)$$

*where the conditional probability*

(16) $$P^{\psi }\left(A=\alpha |B=\beta \right)=P^{\psi_{\beta }}\left(A=\alpha \right)$$

*always refers to the probability of obtaining the outcome α in the A-measurement given that an immediately preceding B-measurement had the outcome β. Analogously,*

(17) $$P^{\psi }\left(B=\beta |A=\alpha \right)=P^{\psi_{\alpha }}\left(B=\beta \right).$$

*Equation *(14)* is thus the joint probability for the outcomes A=α and B=β if the B-measurement precedes the A-measurement, while *(15)* is the joint probability if the A-measurement is performed first. As demonstrated above, it cannot be assumed a priori that these joint probabilities coincide. Instead, we have*

(18) $$P^{\psi }\left(A=\alpha \overset{↼}{\wedge }B=\beta \right)\neq P^{\psi }\left(A=\alpha \vec{\wedge }B=\beta \right)$$

*for an important class of joint measurements (in particular, those described by non-commuting observables). In a relativistic context, this is unproblematic as long as the two measurement events are timelike- or lightlike-related. If the B-measurement occurs in or on the past light-cone of the A-measurement, the relevant distribution is $P^{\psi }\left(A=\alpha \overset{↼}{\wedge }B=\beta \right)$; if the A-measurement occurs in or on the past light-cone of the B-measurement, the relevant distribution is $P^{\psi }\left(A=\alpha \vec{\wedge }B=\beta \right)$.*

The issue becomes much more subtle if the two experiments are performed at spacelike separation. In this case, there is no absolute temporal order between the two measurement events. Which occurs first (or whether they occur simultaneously) depends on the Lorentz frame. This already suggests very strongly that time-order dependent statistics (18) must be excluded for spacelike separated measurements, and we will discuss in more detail why they would indeed imply physical inconsistencies.

Notably, though, the (at least formal) mechanism that can lead to time-order dependent statistics is also operative for spacelike separated measurements on entangled systems: a measurement *A* (with a definite outcome α) on part of an entangled system changes the state in a way that affects probabilities for measurement outcomes B=β on another part of the system. That is, in general,

(19) $$P^{\psi }\left(A=\alpha |B=\beta \right)=P^{\psi_{\beta }}\left(A=\alpha \right)\neq P^{\psi }\left(A=\alpha \right),$$

which is precisely how *nonlocality* is manifested in standard quantum theory.

The physical status of this “change”, i.e., the state reduction $\psi \to \psi_{\beta }$, is beyond the scope of our discussion, which does not presuppose any particular interpretation of the wave function or its “collapse”. Our key assertions can be understood on a purely operational level as pertaining to displayed results of measurements and their statistics predicted by the quantum algorithm.

That said, it is beyond question for the authors that the state reduction must be understood as physically substantial in one way or the other. This is indeed the moral of the EPR argument (together with the violation of Bell’s inequality): Assume the perfect anti-correlations for the spin-singlet state, and consider a frame in which the measurement on particle 1 precedes the measurement on particle 2. Measuring, say, *z*-spin +1 on particle 1, we can infer that a *z*-spin measurement on particle 2 will yield the outcome −1. So, either this outcome was determined in advance (independently of any measurement), or it is, in some way, dynamically determined by the measurement performed on particle 1. Bell’s theorem rules out the first option. Hence, there must be some dynamical influence between the spacelike separated measurement events—even though different observers will disagree on their temporal order and, thus, on the “direction” of the influence (For excellent discussions, see [24,25,26]).

How *this* tension between nonlocality and relativity can be resolved—and the nonlocal correlations explained by a precise Lorentz-invariant quantum theory that solves the measurement problem—is a much harder question. Our goal here is only to identify and justify a precise condition that guarantees consistent statistical predictions across all Lorentz frames.

## 3. Relativistic Consistency

### 3.1. Relativistic Consistency of a Single Quantum Measurement

A basic requirement of relativistic quantum theory is that the Hilbert space associated with the relevant quantum systems carries a unitary representation of the Poincaré group. Here, we will focus on unitary representations U(Λ) of Lorentz boosts Λ.

If one and the same experiment is described in two different Lorentz frames—the lab frame ∑ or a “boosted” frame $\sum^{\prime }=\Lambda \sum$, moving uniformly relative to the lab—several theoretical structures can and will transform in a nontrivial manner. What must remain invariant, however, are the macroscopic records of measurement outcomes, say, the number α=3 displayed on a screen or indicated by a pointer on a scale. From the perspective of $\sum^{\prime }$, the measurement device will appear distorted (by Lorentz contraction), but it must not display a different number (leaving aside purely optical effects such as parallax). Such macroscopic measurement records are the minimal ontological consensus; they (if nothing else) constitute objective facts for which quantum theory yields statistical predictions.

In the lab frame ∑, we denote the prepared initial state by ψ. For an ideal quantum measurement, each possible outcome α is associated with a projection $P_{\alpha }^{A}$. The family ${\left(P_{\alpha }^{A}\right)}_{\alpha }$ corresponds to the spectral decomposition of a self-adjoint operator *A* (“observable”). In the moving frame $\sum^{\prime }$, the initial quantum state takes the form U(Λ)ψ. Operationally, the transformation $\psi \mapsto \psi^{\prime }=U\left(\Lambda \right)\;\psi$ corresponds to a Lorentz boost of the preparation device. The projections are transformed as $P_{\alpha }^{A}\mapsto P_{\alpha }^{A^{\prime }}=U\left(\Lambda \right)\;P_{\alpha }^{A}\;U^{†}\left(\Lambda \right)$ and, accordingly, $A\mapsto A^{\prime }=U\left(\Lambda \right)\;A\;U^{†}\left(\Lambda \right)$. Operationally, this corresponds to a boost of the measuring device.

If the whole experiment performed in the lab is described in $\sum^{\prime }$, i.e., both the preparation device and the measurement device are (passively) boosted, one obtains the following quantum predictions for outcomes $A^{\prime }=\alpha$:

(20) Pψ′(A′=α)=ψ′PαA′ψ′PαA′ψ′ψ′=ψU†(Λ)U(Λ)PαAU†(Λ)U(Λ)ψU†(Λ)U(Λ)PαAU†(Λ)U(Λ)ψψ==ψPαAψPαAψψ=PψA=α.

The unitary representation of Lorentz boosts thus guarantees that we obtain the same statistical predictions whether the experiment is described in ∑ or in $\sum^{\prime }$.

We emphasize again that A=α and $A^{\prime }=\alpha$ refer to the same measurement outcome “α” as recorded by some macroscopic instrument. This is not to be confused with the situation where only the measured system is (actively) boosted, and we observe, for example, a redshifted energy spectrum in the lab frame. Macroscopic measurement records are what they are, independent of the coordinate system or the observer’s state of motion. Accordingly, their statistics (i.e., frequencies) must be the same in all Lorentz frames.

We just saw that, for an individual quantum measurement, a unitary representation of Lorentz boosts is necessary and sufficient for consistent statistical predictions across different Lorentz frames. It is no longer sufficient when we consider joint measurements on entangled systems. As argued before, we must, in addition, require time-order independent statistics if the measurement events are spacelike separated.

The reader may already anticipate that this is closely related to the *local commutativity* of operators, which is commonly taken as an axiom in relativistic quantum (field) theory. This relationship will be discussed in Section 4 (when one goes beyond projective measurements, it is much less trivial than it may seem). First, we continue to discuss the relevant condition on the level of probability distributions, which are closer to the physical content of the theory than abstract operator conditions.

### 3.2. Relativistic Consistency of Spacelike Separated Measurements

Suppose that in the laboratory frame ∑, the *A*-measurement on an entangled system with initial state ψ is followed by a *B*-measurement at spacelike separation. Consider a second Lorentz frame $\sum^{\prime }$ in which the order of the measurements is reversed. Consistent statistical predictions now require

(21) $$P^{\psi }\left(A=\alpha \vec{\wedge }B=\beta \right)\overset{!}{=}P^{\psi^{\prime }}\left(A^{\prime }=\alpha \overset{↼}{\wedge }B^{\prime }=\beta \right),$$

so that observers in both frames agree on the joint probabilities of macroscopic measurement outcomes, even if they disagree on the temporal order of the measurements.

Equation (21) is necessary and sufficient for consistent statistical predictions for measurements outcomes across different Lorentz frames. It is also necessary (though not sufficient) to ensure the consistency of *individual* measurement outcomes. If (21) was violated, predicted relative frequencies of outcomes in the unprimed reference frame would differ from those predicted in the primed frame. If none of the frames are privileged (and the theory empirically adequate), this would entail that if the experiment is repeated sufficiently often, there must be individual runs in which a pointer points to some number (say, “3”) in ∑ but to a different number (say, “7”) in $\sum^{\prime }$. In other words, a violation of (21) would imply inconsistent *facts* in different Lorentz frames.

A unitary representation of Lorentz boosts gets us part of the way. Analogously to (20), they ensure

(22) Pψ′(A′=α∧↼B′=β)==Pψ′(A′=α∣B′=β)Pψ′(B′=β)=Pψβ′(A′=α)Pψ′(B′=β)==ψβU†(Λ)U(Λ)PαAU†(Λ)U(Λ)ψβU†(Λ)U(Λ)PαAU†(Λ)U(Λ)ψβψβ·ψU†(Λ)U(Λ)PβBU†(Λ)U(Λ)ψU†(Λ)U(Λ)PβBU†(Λ)U(Λ)ψψ==ψβPαAψβPαAψβψβ·ψPβBψPβBψψ=Pψβ(A=α)Pψ(B=β)==PψA=α∣B=βPψB=β=Pψ(A=α∧↼B=β).

(Without loss of generality, we can consider Lorentz frames in which the measurements take place in such short intervals that the free time evolution in between is negligible. Assuming that measurement records are stable, the derived identity must then hold in arbitrary frames. See [27] (p. 172) for a more detailed and general derivation).

(22) means that it suffices to require time-order independent statistics in a single Lorentz frame, i.e.,

(23) $$P^{\psi }\left(A=\alpha \overset{↼}{\wedge }B=\beta \right)\overset{!}{=}P^{\psi }\left(A=\alpha \vec{\wedge }B=\beta \right),$$

since (22) and (23) are equivalent to (21).

**Definition** **2** (Relativistic Consistency Condition)**.** *We call*

(24) $$P^{\psi }\left(A=\alpha \overset{↼}{\wedge }B=\beta \right)=P^{\psi }\left(A=\alpha \vec{\wedge }B=\beta \right)$$

*the* relativistic consistency condition, *which must hold for all possible outcomes α and β and any spacelike separated measurements A and B.*

Recalling Definition 1 of the time-ordered joint probabilities, (24) can also be written as

(25) $$P^{\psi }\left(A=\alpha |B=\beta \right)P^{\psi }\left(B=\beta \right)=P^{\psi }\left(B=\beta |A=\alpha \right)P^{\psi }\left(A=\alpha \right)$$

or, equivalently,

(26) $$P^{\psi_{\beta }}\left(A=\alpha \right)\;P^{\psi }\left(B=\beta \right)=P^{\psi_{\alpha }}\left(B=\beta \right)\;P^{\psi }\left(A=\alpha \right).$$

This illustrates again why nonlocality makes relativistic consistency a nontrivial matter. For entangled systems, we generally have

(27) $$P^{\psi }\left(A=\alpha |B=\beta \right)\neq P^{\psi }\left(A=\alpha \right)\;\;\mathrm{and}\;\;P^{\psi }\left(B=\beta |A=\alpha \right)\neq P^{\psi }\left(B=\beta \right).$$

Nonetheless, the products on the left- and right-hand sides of (25) must coincide if nonlocality is not to lead to inconsistencies in a relativistic setting.

### 3.3. From Relativistic Consistency to No-Signaling

Relativistic consistency (24) is the desired condition that ensures the peaceful coexistence of quantum nonlocality and Einsteinian relativity. It is weaker than Bell locality (which is violated by quantum correlations) but stronger than the *no-signaling* condition. That is,

Locality ⇒ Relativistic Consistency ⇒ No-Signaling,

while the converse implications do not hold.

#### 3.3.1. Locality Would Imply Relativistic Consistency (but Not Vice Versa)

Locality (*loc*) implies relativistic consistency since

(28) $$\begin{array}{c} P^{\psi }\left(A=\alpha \overset{↼}{\wedge }B=\beta |\lambda \right)=P^{\psi }\left(A=\alpha |B=\beta ,\;\lambda \right)\;P^{\psi }\left(B=\beta |\lambda \right)\overset{loc}{=}\\ =P^{\psi }\left(A=\alpha |\lambda \right)\;P^{\psi }\left(B=\beta |\lambda \right) \end{array}$$

and analogously

(29) $$\begin{array}{c} P^{\psi }\left(A=\alpha \vec{\wedge }B=\beta |\lambda \right)=P^{\psi }\left(B=\beta |A=\alpha ,\;\lambda \right)\;P^{\psi }\left(A=\alpha |\lambda \right)\overset{loc}{=}\\ =P^{\psi }\left(B=\beta |\lambda \right)\;P^{\psi }\left(A=\alpha |\lambda \right). \end{array}$$

Hence, also $P^{\psi }\left(A=\alpha \overset{↼}{\wedge }B=\beta \right)=P^{\psi }\left(A=\alpha \vec{\wedge }B=\beta \right)$ as one averages (28) and (29) over λ. On the other hand, the standard EPRB example shows that relativistic consistency does not imply locality.

#### 3.3.2. Relativistic Consistency Implies No-Signaling

We now show that (24) implies the no-signaling condition (1). We continue to focus on standard (ideal) measurements before showing in Section 4 that the results extend straightforwardly to more general measurement schemes. For spacelike separated measurements *A* and *B* on a system with initial state ψ, we have

(30) PψB=β∣Awasmeasured=∑αPψB=β∣A=αPψA=α==∑αPψ(A=α∧⇀B=β)=RC∑αPψ(A=α∧↼B=β)==∑αPψA=α∣B=βPψB=β==∑αPψβ(A=α)PψB=β=∑αPβψPαPβψPαPβψPβψ∥Pβψ∥2∥Pβψ∥2==Pβψ∑αPαPβψ∑αPαPβψPβψ=PβψPβψPβψPβψ=∥Pβψ∥2=PψB=β,

where we used that ${\left(P_{\alpha }\right)}_{\alpha }$ is a complete family of projections, i.e., $\sum_{\alpha }P_{\alpha }=1$. Hence, the relativistic consistency condition implies the no-signaling condition (1).

The expression $P^{\psi }\left(B=\beta |A\;\mathrm{was}\;\mathrm{measured}\right)$ is thereby best understood within a Lorentz frame in which the *A*-measurement occurs before the *B*-measurement. $P^{\psi }\left(B=\beta \right)$ can be interpreted in two ways: either as outcome probabilities for the *B*-measurement when no *A*-measurement occurs, or as computed in a frame in which the *A*-measurement occurs afterward (Recall from (20) that $P^{\psi^{\prime }}\left(B^{\prime }=\beta \right)=P^{\psi }\left(B=\beta \right)$ for any Lorentz boost). Under the first interpretation, the no-signaling condition ensures that Alice cannot influence Bob’s outcome statistics by deciding whether or not to perform the *A*-measurement. Since this holds for any *A*, she also cannot influence Bob’s statistics by choosing *which* measurement she performs (e.g., which spin component she measures in an EPRB experiment). Under the second interpretation, where both measurements occur but the left- and right-hand sides refer to different Lorentz frames, (1) is really a weaker relativistic consistency condition necessary for the frame-independence of Bob’s outcome statistics.

#### 3.3.3. No-Signaling Does Not Imply Relativistic Consistency

For a counterexample, consider the following distribution corresponding to an EPR experiment in which the (anti-)correlations hold only in one direction.

$$\begin{array}{ccc} P\left({↑}_{L}\right)=\frac{1}{2}\; & P({↑}_{L}|{↑}_{R})=0\; & {P(↑}_{R}|{↑}_{L})=\frac{1}{2}\\ \;P\left({↓}_{L}\right)=\frac{1}{2}\; & P({↑}_{L}|{↓}_{R})=1\; & {P(↑}_{R}|{↓}_{L})=\frac{1}{2}\\ P\left({↑}_{R}\right)=\frac{1}{2}\; & P({↓}_{L}|{↑}_{R})=1\; & {P(↓}_{R}|{↑}_{L})=\frac{1}{2}\\ P\left({↓}_{R}\right)=\frac{1}{2}\; & P({↓}_{L}|{↓}_{R})=0\; & {P(↓}_{R}|{↓}_{L})=\frac{1}{2} \end{array}$$

We have

$$\begin{array}{c} P^{\psi }{(↑}_{L}\vec{\wedge }{↓}_{R}{)=P(↓}_{R}|{↑}_{L})P\left({↑}_{L}\right)=\frac{1}{2}\cdot \frac{1}{2}=\frac{1}{4}\\ P^{\psi }{(↑}_{L}\overset{↼}{\wedge }{↓}_{R}{)=P(↑}_{L}|{↓}_{R})P\left({↓}_{R}\right)=1\cdot \frac{1}{2}=\frac{1}{2}. \end{array}$$

Hence, $P^{\psi }{(↑}_{L}\vec{\wedge }{↓}_{R})\neq P^{\psi }{(↑}_{L}\overset{↼}{\wedge }{↓}_{R})$ and is a violation of relativistic consistency. And yet, we can verify that the no-signaling conditions are satisfied:

$$\begin{array}{cccc} & {P(↑}_{L}{|↑}_{R})P\left({↑}_{R}\right)+{P(↑}_{L}|{↓}_{R})P\left({↓}_{R}\right)=0\cdot \frac{1}{2}+1\cdot \frac{1}{2} & =\frac{1}{2} & =P({↑}_{L})\;✓\\ & {P(↓}_{L}{|↑}_{R})P\left({↑}_{R}\right)+{P(↓}_{L}|{↓}_{R})P\left({↓}_{R}\right)=1\cdot \frac{1}{2}+0\cdot \frac{1}{2} & =\frac{1}{2} & =P({↓}_{L})\;✓\\ & {P(↑}_{R}{|↑}_{L})P\left({↑}_{L}\right)+{P(↑}_{R}|{↓}_{L})P\left({↓}_{L}\right)=\frac{1}{2}\cdot \frac{1}{2}+\frac{1}{2}\cdot \frac{1}{2} & =\frac{1}{2} & =P({↑}_{R})\;✓\\ & {P(↓}_{R}{|↑}_{L})P\left({↑}_{L}\right)+{P(↓}_{R}|{↓}_{L})P\left({↓}_{L}\right)=\frac{1}{2}\cdot \frac{1}{2}+\frac{1}{2}\cdot \frac{1}{2} & =\frac{1}{2} & =P({↓}_{R})\;✓ \end{array}$$

The example is, of course, an artificial one, since the considered distribution should not be realized in nature—or predicted by quantum theory—for spacelike separated measurements. Although the asymmetric EPR correlations do *not* violate no-signaling conditions, they would entail inconsistent statistics in different Lorentz frames that disagree on the order of the two measurements.

#### 3.3.4. Discussion

As we already complained about in the introduction, the usual motivation for the no-signaling condition is rather anthropocentric. Quantum nonlocality means that there is a sense in which local measurements on entangled systems are influenced by other measurements at spacelike separation. For spacelike separated measurements on entangled systems, we generally have

(31) $$P^{\psi }\left(B=\beta |A=\alpha \right)=P^{\psi_{\alpha }}\left(B=\beta \right)\neq P^{\psi }\left(B=\beta \right),$$

and Bell’s theorem proves that this is not merely a correlation that can be explained by the joint preparation of the system or other “common causes” in the past. The standard argument is that we can live with this nonlocality (even in a relativistic world) because the outcome of the *A*-measurement is (either effectively or fundamentally) random, i.e., not under the control of the experimentalist. What the experimentalist can control is which—if any—measurement *A* to perform. That this decision does not affect the statistics of the distant *B*-measurement means precisely

(32) $$P^{\psi }\left(B=\beta |A\;\mathrm{was}\;\mathrm{measured}\right)=\sum_{\alpha }P^{\psi }\left(B=\beta |A=\alpha \right)P^{\psi }\left(A=\alpha \right)=P^{\psi }\left(B=\beta \right),$$

i.e., the no-signaling condition. The upshot of our analysis is that such anthropocentric arguments are not only questionable but dispensable. The reason why Einsteinian relativity can tolerate nonlocality but not *signaling* is that the latter but not the former implies violations of relativistic consistency. In particular, we show that a violation of the *no-signaling* condition (1) would indeed entail contradictions in a relativistic theory. The reason, however, is not that it would defy some principle of causality or that faster-than-light communication could be used to create paradoxes. The reason is simply that a violation of the *no-signaling* condition implies inconsistent statistical facts in different Lorentz frames.

## 4. Operator Conditions

The goal of this final section is to extend our previous considerations to general quantum measurements and derive operator conditions for relativistic consistency. This will also allow us to address the subtle relationship between the formal condition of *local commutativity* and the more physically transparent concept of relativistic consistency.

### 4.1. Quantum Operations

The most general description of state transformations in standard quantum theory is condensed in the concept of a *quantum operation* W acting on *quantum states*ρ represented by density operators. The states can thus be pure or mixed and *quantum operations* include, in particular, unitary time evolutions, measurement(-like) processes, and the (effective) evolutions of open quantum systems.

Mathematically, a quantum operation is a trace non-increasing, linear, completely positive map acting on density operators (simply put, *complete positivity* means that the map is still positive if the transformed system is described as a subsystem in some larger Hilbert space). Since a density operator ρ has trace 1, its image W(ρ) under a quantum operation W must have a trace in [0,1]. Assuming ${\mathrm{Tr}}_{H}\left[W(\rho )\right]\neq 0$, the resulting state can be normalized to a new density operator

(33) $$\rho^{\prime }=\frac{W(\rho )}{{\mathrm{Tr}}_{H}\left[W(\rho )\right]},$$

which we call the *final state*. In any case, the transition $\rho \to \rho^{\prime }$ occurs with probability

(34) $$P\left(\rho \to \rho^{\prime }\right)={\mathrm{Tr}}_{H}\left[W(\rho )\right].$$

For a detailed exposition of this formalism, see, e.g., [28,29].

In case of unitary transformations, Lindblad-type evolutions for open quantum systems, or non-selective measurements, the transition probability (34) equals 1, i.e., the quantum operation is trace-preserving (and then also called a *quantum channel*). However, our concern is with measurement(-like) processes that result in definite outcomes α and corresponding final states $\rho^{\prime }=\rho_{\alpha }$. These are described by quantum operations $W_{\alpha }$ with nontrivial transition probabilities $P\left(\rho \to \rho_{\alpha }\right)=P^{\rho }\left(\alpha \right)={\mathrm{Tr}}_{H}\left[W_{\alpha }\left(\rho \right)\right]$.

According to the Choi–Kraus theorem [28,30,31], any quantum operation has an operator sum representation (Kraus representation), meaning that there is a set of linear bounded operators $\left\{R_{\alpha k}\right\}$ such that

(35) $$\rho \to \rho_{\alpha }=\frac{\sum_{k=1}^{K_{\alpha }}R_{\alpha k}\;\rho \;R_{\alpha k}^{†}}{{\mathrm{Tr}}_{H}\left[\sum_{k=1}^{K_{\alpha }}R_{\alpha k}\;\rho \;R_{\alpha k}^{†}\right]}=\frac{\sum_{k=1}^{K_{\alpha }}R_{\alpha k}\;\rho \;R_{\alpha k}^{†}}{{\mathrm{Tr}}_{H}\left[\left(\sum_{k=1}^{K_{\alpha }}R_{\alpha k}^{†}\;R_{\alpha k}\right)\;\rho \right]}=:\frac{\sum_{k=1}^{K_{\alpha }}R_{\alpha k}\;\rho \;R_{\alpha k}^{†}}{{\mathrm{Tr}}_{H}\left[E_{\alpha }\;\rho \right]}.$$

The operators $E_{\alpha }:=\sum_{k=1}^{K_{\alpha }}R_{\alpha k}^{†}\;R_{\alpha k}$ (where $K_{\alpha }$ may also be 1 or *∞*) are called *effects*. They are positive (and thus also self-adjoint) operators bounded from above by 1. In view of (34), the state transformation (35) corresponding to the outcome α is realized with probability

(36) $$P^{\rho }\left(\alpha \right)={\mathrm{Tr}}_{H}\left[E_{\alpha }\;\rho \right].$$

The map $\alpha \mapsto E_{\alpha }$ generates a *positive operator valued measure (POVM)* with normalization $\sum_{\alpha }E_{\alpha }=1$ (corresponding to the normalization of the associated probabilities). For any ρ, the POVM then defines a probability distribution over possible outcomes by (36).

For simplicity, we consider here only discrete measurements, i.e., assume that the set of possible outcomes {α} is countable. On the level of POVMs, the generalization to continuous measurements is standard, but the corresponding state transformations are usually unclear. If there exists a corresponding completely positive state transformation (whatever it may look like), the Choi–Kraus theorem still applies and provides a countable operator sum representation. That said, we believe that all physical measurements are actually discrete, while continuous measurements are merely mathematical idealizations.

#### Efficient Measurements

For simplicity, we shall focus on so-called *efficient measurements*, which means that the associated state transformations (35) map pure initial states to pure final states. This is the case if and only if each outcome α is associated with a single state transformation operator $R_{\alpha }$ (that is, $K_{\alpha }=1$ for all α). Hence, we can drop in (35) the sums over *k*. This makes it easier to see the connection to the textbook formalism and our discussion in the previous sections. Generalizing to non-efficient measurements is straightforward by the linearity of the convex sums.

For efficient measurements, we can write down the analog of (35) for pure initial states ψ∈H:

(37) ψ↦ψα=Rαψ∥Rαψ∥=RαψψRα†RαψRα†Rαψψ=RαψψEαψEαψψ.

The corresponding transition probability is

(38) Pψα=∥Rαψ∥2=ψEαψEαψψ,withEα=Rα†Rα.

By polar decomposition (recalling that $E_{\alpha }$ is a positive operator), the *state transformer* $R_{\alpha }$ can be written as

(39) $$R_{\alpha }=U_{\alpha }\sqrt{E_{\alpha }},$$

where $U_{\alpha }$ is a partial isometry. When $U_{\alpha }=1$ and $E_{\alpha }=P_{\alpha }$ is an orthogonal projection ($E_{\alpha }^{2}=E_{\alpha }=\sqrt{E_{\alpha }}$), we recover the ideal measurement scheme with

(40) $$R_{\alpha }\equiv E_{\alpha }\equiv P_{\alpha },$$

and the POVM defined by the projections $\left\{P_{\alpha }\right\}$ is a projection-valued measure (PVM) corresponding to a self-adjoint observable $A=\sum_{\alpha }\;\alpha \;P_{\alpha }$.

Note, however, that not all projective measurements are ideal measurements. The state transformers $R_{\alpha }=U_{\alpha }P_{\alpha }$ of a projective measurement can still involve, e.g., a unitary rotation $U_{\alpha }\neq 1$ (sometimes called “measurement back action”). A projective measurement is called *reproducible* iff $U_{\alpha }$ leaves the eigenspaces $P_{\alpha }H$ invariant, meaning that the measurement will yield the same outcome upon immediate repetition.

Table 1 gives a rough overview of important classes of efficient measurements (generalizations to the non-efficient case are to be made in the obvious way).

The general measurement formalism is particularly relevant for two reasons. First, modern experimental techniques, such as indirect measurements, are, in general, non-projective. Second, a careful analysis of the measurement process suggests that properties like projectivity or reproducibility are never exactly realized in real-world experiments (at least if the quantum nature of the measuring device is taken into account, see [22,32]). The general measurement formalism can even be derived from such an analysis without the need to postulate operators (ibid.; see also [33]).

### 4.2. Time Order and Relativistic Consistency

Let us consider again the situation of an *A*-measurement followed (in a given Lorentz frame) by a *B*-measurement. If the *A*-measurement results in the outcome α, the state transforms according to (37), i.e., $\psi \mapsto \psi_{\alpha }=\frac{R_{\alpha }^{A}\;\psi }{∥R_{\alpha }^{A}\;\psi ∥}$. The conditional probability of obtaining B=β in the subsequent measurement is thus

(41) PψB=β∣A=α=Pψα(B=β)=ψ(RαA)†EβBRαAψ(RαA)†EβBRαAψψ∥RαAψ∥2.

We see that the joint probabilities for A=α and B=β, when the *A*-measurement occurs first, are encoded in the operator ${\left(R_{\alpha }^{A}\right)}^{†}E_{\beta }^{B}\;R_{\alpha }^{A}$. More precisely,

(42) Pψ(A=α∧⇀B=β)=PψB=β∣A=αPψA=α==ψ(RαA)†EβBRαAψ(RαA)†EβBRαAψψ∥RαAψ∥2∥RαAψ∥2=ψ(RαA)†EβBRαAψ(RαA)†EβBRαAψψ.

Analogously, we have

Pψ(A=α∧↼B=β)=ψ(RβB)†EαARβBψ(RβB)†EαARβBψψ.

A Lorentz boost Λ acts on states and operators (effects and state transformers) as $\psi \overset{\Lambda }{\to }U\left(\Lambda \right)\psi$, $E_{\alpha }^{A}\overset{\Lambda }{\to }U\left(\Lambda \right)E_{\alpha }^{A}U^{†}\left(\Lambda \right)$, and $R_{\alpha }^{A}\overset{\Lambda }{\to }U\left(\Lambda \right)R_{\alpha }^{A}U^{†}\left(\Lambda \right)$. In analogy to (20)–(22), we can thus derive the relativistic consistency condition

(43) $$P^{\psi }\left(A=\alpha \overset{↼}{\wedge }B=\beta \right)=P^{\psi }\left(A=\alpha \vec{\wedge }B=\beta \right)$$

for spacelike separated measurements, for which we now obtain explicit expressions

(44) ψ(RβB)†EαARβBψ(RβB)†EαARβBψψ=ψ(RαA)†EβBRαAψ(RαA)†EβBRαAψψ.

Since this must hold for all ψ∈H and the operators ${\left(R_{\beta }^{B}\right)}^{†}E_{\alpha }^{A}\;R_{\beta }^{B}$ and ${\left(R_{\alpha }^{A}\right)}^{†}E_{\beta }^{B}\;R_{\alpha }^{A}$ are self-adjoint, the identity must hold between the operators themselves. We thus obtain the following:

**Definition** **3** (Relativistic Consistency Operator Conditions)**.** *The conditions*

(45) $${\left(R_{\beta }^{B}\right)}^{†}E_{\alpha }^{A}\;R_{\beta }^{B}={\left(R_{\alpha }^{A}\right)}^{†}E_{\beta }^{B}\;R_{\alpha }^{A}$$

*are the relativistic consistency operator conditions. They must hold for all α and β whenever the A- and B-measurements are spacelike separated to guarantee relativistic consistency of the statistical predictions.*

### 4.3. No-Signaling

Summing (45) over α and β, respectively, yields

(46) $$\begin{array}{c} \sum_{\beta }{\left(R_{\beta }^{B}\right)}^{†}E_{\alpha }^{A}\;R_{\beta }^{B}\overset{RC}{=}\sum_{\beta }{\left(R_{\alpha }^{A}\right)}^{†}E_{\beta }^{B}\;R_{\alpha }^{A}={\left(R_{\alpha }^{A}\right)}^{†}\underset{=1}{\underset{︸}{\left(\sum_{\beta }E_{\beta }^{B}\right)}}\;R_{\alpha }^{A}={\left(R_{\alpha }^{A}\right)}^{†}\;R_{\alpha }^{A}=E_{\alpha }^{A},\\ \sum_{\alpha }{\left(R_{\alpha }^{A}\right)}^{†}E_{\beta }^{B}\;R_{\alpha }^{A}\overset{RC}{=}\sum_{\alpha }{\left(R_{\beta }^{B}\right)}^{†}E_{\alpha }^{A}\;R_{\beta }^{B}={\left(R_{\beta }^{B}\right)}^{†}\underset{=1}{\underset{︸}{\left(\sum_{\alpha }E_{\alpha }^{A}\right)}}\;R_{\beta }^{B}={\left(R_{\beta }^{B}\right)}^{†}\;R_{\beta }^{B}=E_{\beta }^{B}. \end{array}$$

These are precisely the no-signaling conditions in operator form, since they are necessary and sufficient for

(47) PψA=α=ψEαAψEαAψψ=(46)ψ∑β(RβB)†EαARβBψ∑β(RβB)†EαARβBψψ=∑βψ(RβB)†EαARβBψ(RβB)†EαARβBψψ==∑βψ(RβB)†EαARβBψ(RβB)†EαARβBψψψEβBψEβBψψψEβBψEβBψψ=∑βPψA=α∣B=βPψB=β==PψA=α∣Bwasmeasured

and analogously for the marginal distribution of the *B*-measurement outcomes. The operator conditions for *relativistic consistency* thus imply the operator conditions for *no-signaling*.

**Definition** **4** (No-Signaling Operator Conditions)**.** *The conditions*

(48) $$\begin{array}{c} E_{\alpha }^{A}=\sum_{\beta }{\left(R_{\beta }^{B}\right)}^{†}E_{\alpha }^{A}\;R_{\beta }^{B}\;\;\mathrm{for}\;\mathrm{all}\;\alpha \\ E_{\beta }^{B}=\sum_{\alpha }{\left(R_{\alpha }^{A}\right)}^{†}E_{\beta }^{B}\;R_{\alpha }^{A}\;\;\mathrm{for}\;\mathrm{all}\;\beta \end{array}$$

*are the no-signaling operator conditions. They guarantee that quantum nonlocality cannot be used to send superluminal signals by acts of measurement.*

For completeness, we state the general form of the operator conditions that also apply to mixed states and non-efficient measurements. Their detailed derivation can be found in [27].

**Definition** **5** (Operator Conditions, General Form)**.**

*(a)*  *The general form of the* relativistic consistency operator conditions *is*
  (49) $$\sum_{l=1}^{L_{\beta }}{\left(R_{\beta ,l}^{B}\right)}^{†}\;E_{\alpha }^{A}\;R_{\beta ,l}^{B}=\sum_{k=1}^{K_{\alpha }}{\left(R_{\alpha ,k}^{A}\right)}^{†}\;E_{\beta }^{B}\;R_{\alpha ,k}^{A}$$
  *for all α and β, whenever the A- and B-measurements are spacelike separated.*
*(b)*  *The general form of the* no-signaling operator conditions *is*
  (50) $$\begin{array}{c} E_{\alpha }^{A}=\sum_{\beta }\sum_{l=1}^{L_{\beta }}{\left(R_{\beta ,l}^{B}\right)}^{†}\;E_{\alpha }^{A}\;R_{\beta ,l}^{B}\\ E_{\beta }^{B}=\sum_{\alpha }\sum_{k=1}^{K_{\alpha }}{\left(R_{\alpha ,k}^{A}\right)}^{†}\;E_{\beta }^{B}\;R_{\alpha ,k}^{A} \end{array}$$
  *for all α and all β, respectively.*

### 4.4. Local Commutativity

From (45), we also begin to see what *local commutativity* has to do with *relativistic consistency*. The relativistic consistency operator conditions are automatically satisfied if the effects of a local measurement commute with the state transformers of any spacelike separated measurement. That is,

(51) $$\left[E_{\alpha }^{A},R_{\beta }^{B}\right]=\left[E_{\beta }^{B},R_{\alpha }^{A}\right]=0\;\mathrm{for}\;\mathrm{all}\;\alpha ,\beta$$

whenever *A* and *B* are spacelike separated. Since then,

(52) $$\begin{array}{c} {\left(R_{\beta }^{B}\right)}^{†}E_{\alpha }^{A}\;R_{\beta }^{B}\overset{\left(51\right)}{=}{\left(R_{\beta }^{B}\right)}^{†}R_{\beta }^{B}\;E_{\alpha }^{A}=E_{\beta }^{B}\;{\left(R_{\alpha }^{A}\right)}^{†}R_{\alpha }^{A}=\\ ={\left(R_{\alpha }^{A}\;E_{\beta }^{B}\right)}^{†}R_{\alpha }^{A}\overset{\left(51\right)}{=}{\left(E_{\beta }^{B}\;R_{\alpha }^{A}\right)}^{†}\;R_{\alpha }^{A}={\left(R_{\alpha }^{A}\right)}^{†}E_{\beta }^{B}\;R_{\alpha }^{A}. \end{array}$$

(Based on similar considerations, ref. [16] lands on a stronger commutativity condition—the local commutativity of state transformers, i.e., $[R_{\alpha }^{A},R_{\beta }^{B}]=0$ in our notation—which is also sufficient for relativistic consistency). We further see that (51) implies

(53) $$\left[E_{\alpha }^{A},E_{\beta }^{B}\right]=\left[E_{\beta }^{B},E_{\alpha }^{A}\right]=0\;\mathrm{for}\;\mathrm{all}\;\alpha ,\beta ,$$

i.e., the local commutativity of effects. For projective measurements, where $\left\{E_{\alpha }^{A}\right\}$ and $\left\{E_{\beta }^{B}\right\}$ are spectral decompositions of self-adjoint observables $\hat{A}$ and $\hat{B}$, (53) is equivalent to $[\hat{A},\hat{B}]=0$. Notably, though, (53) does not imply (51), which is hence the more general local commutativity condition (Indeed, an effect need not even commute with its own state transformer, while it trivially commutes with itself: Consider, for example, a projective measurement with outcomes α, orthogonal projections Eα=αααα, and state transformers Rα=ωααω=ωααωαααα≡UαEα with partial isometries Uα=ωααω. This can be seen as a toy model of a measurement in which the particle is absorbed after producing the outcome α, which is implemented by mapping all states into the vacuum |ω〉 orthogonal to all |α〉. We have $R_{\alpha }^{†}R_{\alpha }=E_{\alpha }$ but [Eα,Rα]=−ωααω=−Rα≠0).

Since some of the literature refers to local commutativity as “locality”, we also emphasize that it must not be confused with locality in the sense of Bell’s theorem. Indeed, the EPRB experiment indicates a violation of Bell locality, even though the spin observables for different particles trivially commute.

So far, we have in terms of operator conditions:

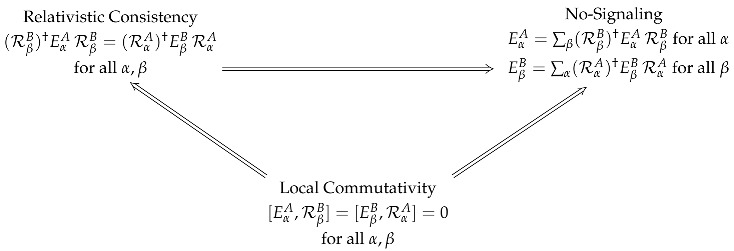
(54)

As a mathematical postulate, local commutativity is the most practical choice. It is often justified by appealing to no-signaling, either explicitly or implicitly, by saying something like “a measurement should not be disturbed by others occurring at spacelike separation” (Of course, they *are* disturbed in the sense of nonlocality, just not in a way that affects their marginal outcome distributions). However, as argued throughout this paper, the relativistic consistency condition is the one that can be physically justified from first principles.

It would be nice if one could close the circle in (54), i.e., show that the no-signaling operator conditions imply local commutativity, so that all three conditions—local commutativity, relativistic consistency, and no-signaling—turn out to be equivalent on the level of operators. This would mean, in particular, that probability distributions which satisfy no-signaling but violate relativistic consistency cannot arise from quantum operations. The issue is, however, a subtle one.

A very general result about the equivalence of local commutativity and no-signaling is the following theorem (see [34] for a proof). For any effect $E_{\beta }^{B}$ and any set of state transformers $\left\{R_{\alpha }^{A}\right\}$, the following two conditions are equivalent:

(i) *Commutativity of effect and state transformers:*
  (55) $$\left[E_{\beta }^{B},R_{\alpha }^{A}\right]=\left[E_{\beta }^{B},{\left(R_{\alpha }^{A}\right)}^{†}\right]=0\;\mathrm{for}\;\mathrm{all}\;\alpha$$
(ii) *No-signaling condition for $E_{\beta }^{B}$ and ${\left(E_{\beta }^{B}\right)}^{2}$ with respect to $\left\{R_{\alpha }^{A}\right\}$:*
  (56) $$E_{\beta }^{B}=\sum_{\alpha }{\left(R_{\alpha }^{A}\right)}^{†}E_{\beta }^{B}\;R_{\alpha }^{A}\;\;\mathrm{and}\;\;{\left(E_{\beta }^{B}\right)}^{2}=\sum_{\alpha }{\left(R_{\alpha }^{A}\right)}^{†}{\left(E_{\beta }^{B}\right)}^{2}\;R_{\alpha }^{A}$$

This implies, in particular, the equivalence of local commutativity and no-signaling for projective measurements (Lüders theorem), since ${\left(E_{\beta }^{B}\right)}^{2}=E_{\beta }^{B}$ if the POVM elements are projectors.

The theorem settles the equivalence of no-signaling and local commutativity altogether if one takes for granted—as algebraic approaches (e.g., [35]) implicitly do—that the squared effects ${\left(E_{\beta }^{B}\right)}^{2}$ correspond to some local measurement *C* in the same spacetime region as *B*. In this case, no-signaling entails both conditions in (56) anyway if the *A*-measurement is spacelike separated from the *B* and *C* measurements. The problem is that the physical interpretation of the squared effects ${\left(E_{\beta }^{B}\right)}^{2}$ is, in general, unclear, and it is doubtful that they always correspond to some meaningful measurement-like process (This is different for common observables $B=\sum_{\beta }\;\beta \;P_{\beta }$, where $B^{2}=\sum_{\beta }\;\beta^{2}\;P_{\beta }$ can simply be associated with the same measurement process as *B* by rescaling the outcome values according to $\beta \mapsto \beta^{2}$). We can still impose no-signaling conditions for them—or for entire local algebras of operators while we are at it—but why should operators have to satisfy no-signaling conditions if they do not correspond to any possible process or event that could occur in nature?

If the state transformers $R_{\beta }^{B}=U_{\beta }^{B}\sqrt{E_{\beta }^{B}}$ are normal operators (commute with their adjoints), ${\left(E_{\beta }^{B}\right)}^{2}$ has an immediate physical interpretation as the effect associated with obtaining the outcome β twice if the *B*-measurement is immediately repeated. Indeed, the corresponding effect would be

(57) $${\left(R_{\beta }^{B}\right)}^{†}E_{\beta }^{B}\;R_{\beta }^{B}={\left(R_{\beta }^{B}\right)}^{†}{\left(R_{\beta }^{B}\right)}^{†}R_{\beta }^{B}R_{\beta }^{B}={\left(R_{\beta }^{B}\right)}^{†}R_{\beta }^{B}{\left(R_{\beta }^{B}\right)}^{†}R_{\beta }^{B}={\left(E_{\beta }^{B}\right)}^{2}.$$

But not all measurements have normal state transformers (for an instructive counterexample, see the particle absorption toy model with Rα=ωααω in the remark above). It might even be that no real-world measurements actually do and that all “textbook measurements” are merely idealizations. Certainly, many real-world experiments can be described by normal state transformers or projective effects *for all practical purposes* (FAPP). However, if one appeals to rigorous mathematical results (in order to justify the physical status of local commutativity), FAPP may not be good enough.

Several other results proving the necessity of local commutativity for no-signaling and/or relativistic consistency for certain classes of operators can be found in Chapter 3 of [27] and the references therein. The problem remains that the class of operators corresponding to “local measurement-like processes that could occur in nature” eludes a precise mathematical definition, so there is always the risk of assuming too much or proving too little.

On the other hand, there exist formal counterexamples to the implication $E=\sum_{\alpha }{\left(R_{\alpha }\right)}^{†}\;E\;R_{\alpha }\Rightarrow \;\left[E,R_{\alpha }\right]=0\;\forall \alpha$ (e.g., [34,36]), but their physical significance is at least equally questionable. They are, moreover, only partial counterexamples to the implication No-Signaling⇒LocalCommutativity. A full (formal) counterexample would require two complete sets of state transformers $\left\{R_{\alpha }^{A}\right\},\;\left\{R_{\beta }^{B}\right\}$, and corresponding POVMs $\left\{E_{\alpha }^{A}\right\},\left\{E_{\beta }^{B}\right\}$ such that all no-signaling conditions (48) are satisfied, while (51) is violated for at least one pair of outcomes α,β.

While this discussion hardly exhausts the topic, we hope it makes clear that local commutativity is, in the first place, an abstract condition whose mathematical convenience tends to obscure the fact that it has no direct physical interpretation. This is in contrast to the relativistic consistency condition, whose physical meaning is simply that joint statistics for spacelike separated measurements must be consistent across different Lorentz frames. The best justification for postulating local commutativity in a relativistic quantum theory is that it is at least sufficient and, for many classes of relevant measurements, also necessary for relativistic consistency. In any case, from a physical and conceptual point of view, relativistic consistency (rather than local commutativity or no-signaling) should be understood as the fundamental condition for a peaceful coexistence of quantum theory and special relativity.

## Figures and Tables

**Table 1:** Some classes of efficient measurements. Conditions are meant to hold for all α (unless stated otherwise).

Projective Measurements		
Ideal	$R_{\alpha }=P_{\alpha }$	$U_{\alpha }=1$
Reproducible	$R_{\alpha }=U_{\alpha }\;P_{\alpha }$	$U_{\alpha }P_{\alpha }H=P_{\alpha }H$
Non-Reproducible	$R_{\alpha }=U_{\alpha }\;P_{\alpha }$	$\exists \alpha :U_{\alpha }P_{\alpha }H\neq P_{\alpha }H$
**Non-Projective Measurements**		
Lüders Measurements	$R_{\alpha }=\sqrt{E_{\alpha }}$	$U_{\alpha }=1$
General Measurements	$R_{\alpha }=U_{\alpha }\sqrt{E_{\alpha }}$	$U_{\alpha }$

## Data Availability

No new data were created or analyzed in this study. Data sharing is not applicable to this article.
